# DOES MALE GENDER INCREASE THE RISK OF LAPAROSCOPIC CHOLECYSTECTOMY?

**DOI:** 10.1590/0102-672020190001e1438

**Published:** 2019-08-26

**Authors:** Júlio Cezar Uili COELHO, Giuliano Ohde DALLEDONE, Wagner SCHIEL, Jacqueline de Pauli BERBARDIN, Christiano M. P. CLAUS, Jorge E.F. MATIAS, Alexandre C. T. de FREITAS

**Affiliations:** 1 Surgical Service of the Digestive System, Our Lady of Grace Hospital; 2 Discipline of Clinical Surgery, Federal University of Paraná, Curitiba, PR, Brazil.

**Keywords:** Cholelithiasis, Laparoscopic cholecystectomy, Postoperative complication, Gallstone, Cholecystitis, Colelitíase, Colecistectomia laparoscópica, Complicação pós-operatória, Cálculo biliar, Colecistite

## Abstract

***Background*:**

Laparoscopic cholecystectomy is the preferable treatment for chronic or acute cholecystitis. Some factors may increase the rate of laparoscopic conversion to open cholecystectomy and perioperative complications. The role of gender as a risk factor for laparoscopic cholecystectomy is controversial.

***Aim*:**

To evaluate the role of the gender on the operative findings and outcome of laparoscopic cholecystectomy.

***Method*:**

All patients who underwent laparoscopic cholecystectomy for chronic or acute cholecystitis were included. Demographic, clinical, laboratory, imaging exams, intraoperative and postoperative data were obtained and analyzed. The data was obtained retrospectively from electronic medical records and study protocols.

***Results*:**

Of a total 1,645 patients who were subjected to laparoscopic cholecystectomy, 540 (32.8%) were men and 1,105 (67.2%) were women. Mean age was similar in both genders (p=0.817). Operative time has longer in the male (72.48±28.50) than in the female group (65.46±24.83, p<0.001). The rate of acute cholecystitis was higher in the male (14.3%) than in the female group (5.1%, p<0.001). There was no difference between the genders in regard to the rate of conversion (p=1.0), intraoperative complication (p=1.0), postoperative complication (p=0.571), and operative mortality (p=1.0).

***Conclusion*:**

Male gender is not an independent risk factor for laparoscopic conversion and perioperative complications.

## RESUMO

*****Racional***:**

A colecistectomia laparoscópica é o tratamento de escolha para colecistite crônica ou aguda. Alguns fatores podem aumentar a taxa de conversão para colecistectomia laparotômica e de complicações perioperatórias. O papel do gênero, como um fator de risco para colecistectomia laparoscópica, é controverso.

***Objetivo*:**

Avaliar o papel do gênero nos achados operatórios e no desfecho da colecistectomia laparoscópica.

***Métodos*:**

Todos os pacientes que foram submetidos à colecistectomia laparoscópica por colecistite crônica ou aguda foram incluídos. Dados demográficos, clínicos, laboratoriais, de imagem, intraoperatórios e pós-operatórios foram obtidos e analisados. Os dados foram obtidos retrospectivamente a partir de prontuários eletrônicos e protocolos de estudo.

***Resultados*:**

De um total de 1.645 pacientes que foram submetidos à colecistectomia laparoscópica, 540 (32,8%) eram homens e 1.105 (67,2%) mulheres. A idade média foi semelhante em ambos os gêneros (p=0,817). O tempo operatório foi maior nos homens (72,48±28,50) do que nas mulheres (65,46±24,83) (p<0,001). A taxa de colecistite aguda foi maior no grupo masculino (14,3%) do que no feminino (5,1%, p<0,001). Não houve diferença entre os gêneros quanto à taxa de conversão (p=1,0), complicação intraoperatória (p=1,0), complicação pós-operatória (p=0,571) e mortalidade operatória (p=1,0).

***Conclusão*:**

O gênero masculino não é fator de risco independente para a conversão laparoscópica e complicações perioperatórias.

## INTRODUCTION

Gallstone is one of the most common diseases, with a prevalence of about 10% in the general population of Brazil and in most western countries^6^ ^,^ ^9^ ^,^ ^10^. 

After the first laparoscopic cholecystectomy (LC) performed by Mühe in Germany in 1986, it became rapidly the “gold standard” treatment for symptomatic gallstone disease^18^. LC is one of the most common surgical procedures worldwide. Almost one million of cholecystectomies are performed annually in the United States, 90% of which are through the laparoscopic access^24^.

LC is associated with several advantages, including minimal trauma, rapid recovery, less pain, better esthetic results, less overall cost, and low rate of postoperative complications^17^ ^,^ ^20^ ^,^ ^22^. Some risk factors such as acute cholecystitis, obesity, and age have been associated with higher rate of laparoscopic conversion to open cholecystectomy, morbidity, and mortality^7^ ^,^ ^11^ ^,^ ^12^. Although some authors have reported that male gender may also be a risk factor for LC complications, the role of the gender in the rate laparoscopic conversion and complications in LC has not been clearly elucidated^2^ ^,^ ^13^ ^,^ ^21^. 

This subject has not been evaluated in Brazil. The objective of our study is to assess the operative findings and outcome of CL in male gender in a Brazilian teaching hospital.

## METHODS

The present study was approved by the Ethical Committee of the Hospital de Clínicas of the Federal University of Parana (Protocol approval number 3.037.086). All patients who underwent laparoscopic cholecystectomy for symptomatic gallstone, either chronic or acute cholecystitis, in our surgical unit at the Hospital Nossa Senhora das Graças, Curitiba, Brazil from January 1, 2011 to March 31, 2018 were included in the study. 

Indications for cholecystectomy were history or presence of biliary colic, jaundice, cholangitis, or biliary pancreatitis. In all cases, diagnosis of gallbladder stones was established by ultrasonography. As a protocol in our surgical unit, all patients with chronic or acute cholecystitis were initially considered for laparoscopic procedure. Critically ill patients with acute cholecystitis with high risk for cholecystectomy underwent percutaneous transparietohepatic cholecystostomy and were excluded from the study.

Other criteria for exclusion from the study were patients who underwent cholecystectomy for neoplasia and the ones who were subjected to an additional surgical procedure, except umbilical hernia repair and liver biopsy. Patients with presence or history of acute pancreatitis, jaundice, and dilation of the common bile duct on ultrasonography were subjected to magnetic resonance cholangiography. If common bile duct stones were identified, retrograde cholangiopancreatography and endoscopic stone extraction were performed, and the patients were also excluded from the study.

All operations were performed or supervised by the same surgeon. Surgical residents have participated of all operations. LC has been performed by our group since 1991.

After routine temporary nasogastric tube insertion, the abdominal cavity was insufflated with CO_2_ at an intrabdominal pressure of ≤14 mmHg. Four trocars, two of 5 mm and two of 10 mm were carefully inserted into the abdominal cavity. Operative cholangiography was performed only in selected cases, i.e. dilation of common bile duct, difficulty of identification of biliary tree anatomy, and suspicion of biliary tree lesion. Immediately prior to wound closure at the end of the operation, all layers of all four surgical incisions were infiltrate with local anesthetic (bupivacaine 0.5%). The patients received a single intra-operative dose of intravenous parocoxib sodium 40 mg, tramadol hydrochloride 100 mg, and dipyrone 2 g for analgesia. A single dose of 4 mg of ondansetron was also administered intravenously prior to completion of the procedure to prevent postoperative nauseas and vomiting. 

Patients returned for ambulatory follow-up at the 7^th^ day, and one and three months after operation. Follow-up was extended as needed in presence of complications. 

The following data were obtained and analyzed: age, gender, clinical and diagnostic tests findings, American Society of Anesthesiology score (ASA), operative findings and complications, type of operation, postoperative complications and mortality, hospital stay duration, and hospital readmission. Indications for conversion to open cholecystectomy were also recorded. The data was obtained retrospectively from electronic medical records and study protocols.

### Statistical analysis

 Values were expressed as mean± SD (standard-deviation). Statistical analysis was performed using the IBM SPSS Statistics version 23.0 program (IBM Inc., Armonk, NY, USA). Student´s t-test was employed to determine the difference between the means and chi-square test to assess the difference between the expected frequencies and the observed frequencies of the two groups. Results with p-value ≤0.05 (5%) were considered as statistically significant.

## RESULTS

Of a total 1,645 patients who were subjected to laparoscopic cholecystectomy, 540 (32.8%) were men and 1,105 (67.2%) women. The female to male rate was 2:1. Table 1 shows the comparison of demographic and clinical characteristics of the two groups. Mean age was similar for male (52.17±14.33) and female (49.26±15.85, p=0.817). The clinical presentation of both groups was biliary colic, fever and/or jaundice. Jaundice was more common in men than in women (p=0.011). The rate of prior abdominal operation was similar in both groups (p=0.417).

**TABLE 1:** Demographic and clinical characteristics

Characteristics	Men n (%)	Women n (%)	p
Number	540 (32.8%)	1,105 (67.2%)	
Age (years) Range mean ± SD	15-94 52.17 ± 14.33	12-100 49.26 ±15.85	0.817
Clinical presentation Biliary colic Fever Jaundice	540 (100) 7 (1.3) 8 (1.5)	1,105 (100) 2 (0.2) 5 (0.5)	1.0 0.419 0.011
Prior abdominal surgery	106 (19.6%)	193 (17.5%)	0.417
ASA Score I II III IV	243 (45.0) 287 (53.1) 10 (1.9) 0	527 (47.7) 562 (50.9) 15 (1.4) 1 (0.1)	0.562 0.376 0.442 0.294

Preoperative American Society of Anesthesiologists (ASA) score distribution of patients of the two groups is shown in Table 1. Most patients of both groups had score I or II. There was no difference in the distribution of ASA scores between male and female (score I, p=0.562; score II, p=0.376; score III, p=0.442; score IV, p=0.294). 

Table 2 shows the intraoperative and postoperative data. Operative time has longer in the male (72.48±28.50) than in the female group (65.46±24.83, p<0.001). The rate of acute cholecystitis was higher in the male (16.30%) than in the female group (7.24%, p<0.001). The conversion rate to open cholecystectomy was similar in both groups (p=1.0). The single conversion in the male group was due to difficulty to identify the biliary tract anatomy due to intense gallbladder fibrosis and adherence to adjacent structures. The causes for conversion in women were failure to adequately identify the biliary tract anatomy due to intense gallbladder fibrosis and adherence to adjacent structures (n= 2), uncontrolled intraoperative bleeding by laparoscopy (n=1) and lesion of the transverse colon during insertion of the trocar in a patient with intense abdominal adhesions due to prior abdominal operation (n=1).

**TABLE 2:** Intraoperative and postoperative data

Characteristics	Men 540 (32.8%)	Women 1,105 (67.2%)	p
Operative time (min) Range mean ± SD	25 to 225 72.48 ± 28.50	25 to 220 65.46 ± 24.83	<0.001
Acute cholecystitis	88 (16.30%)	80 (7.24%)	<0.001
Conversion to open cholecystectomy	1 (0.19%)	4 (0.36%)	1.0
Intraoperative complications	4 (0.74%)	2 (0.18%)	0.770
Postoperative complications	32 (5.93%)	58 (5.24%)	0.571
Postoperative mortality	1 (0.19%)	2 (0.18%)	1.0
Hospital stay length (days)	1.14 ± 1.14	1.07 ± 0.88	0.206

There was no difference in the rate of intraoperative complications between the two groups (p=0.770). Intraoperative complications of the male group were severe bronchospasm at extubation (n=2), lesion of the right hepatic artery (n=1), and small bowel perforation (n=1). Intense bleeding due to liver laceration (n=1) and colonic perforation (n=1) occurred in the female group.

Postoperative complication rate was similar in men (5.93%) and in women (5.24%, p=0.571). Table 3 displays the postoperative complications of the two groups. The most common complications in both age groups were related with the wound at the umbilicus, namely hematoma, infection, and incision hernia.

**TABLE 3:** Postoperative complications*

Complication	MEN n (%)	WOMEN n (%)
Surgical site infection	5 (15.6)	6 (10.3)
Pulmonary atelectasis	4 (12.5)	9 (15.5)
Incisional hernia	3 (9.4)	7 (12.7)
Subcutaneous hematoma	2 (6.3)	6 (10.3)
Venous thrombosis	2 (6.3)	7 (12.7)
Subhepatic abscess	2 (6.3)	2 (3.4)
Urinary retention	5 (15.6)	3 (5.2)
Urinary infection	1 (3.1)	2 (3.4)
Pneumonia	1 (3.1)	2 (3.4)
Cardiac arrhythmia	2 (6.3)	2 (3.4)
Biliary fistula	1 (3.1)	1 (1.7)
Intestinal fistula	0	1 (1.7)
Skin burning	0	1 (1.7)
Others	4 (12.5)	9 (15.5)
Total	32	58

*Some patients had more than one complication

Four patients, two of each group, presented with fever and abdominal pain and loss of appetite. Following identification of subhepatic abscess by tomography, the collections were successfully treated with broad-spectrum intravenous antibiotics in two patients and ultrasound-guided percutaneous drainage and parenteral antibiotics in the other two. Biliary fistula was diagnosed in one patient of each group. Both with biliary fistula presented with subhepatic fluid collection which were treated conservatively with ultrasound-guided percutaneous tube drainage. 

Operative mortality was similar in men (0.19%) and in women (0.18%) (p=1.0). One patient (0.19) died from myocardial infarction in the male group. Two patients (0.18%) died in women group, one from pneumonia and other from *Pseudomonas* sepsis following embolectomy and fasciotomy due to postoperative tibial artery embolism. There is no difference in hospital stay length between the two groups (p=0.206).

## DISCUSSION

LC became the gold standard procedure to treat symptomatic gallstone in few years after its introduction due to its multitude of advantages. Although its rate of perioperative complications is less than open cholecystectomy, severe complications may occur^3^. The rate of biliary tree lesion is higher in patients subjected to LC than open cholecystectomy. In addition, some patients are subjected to conversion from LC to open surgery due to technical difficulties to identify the anatomy^8^ ^,^ ^14^. 

Although laparoscopic conversion to open surgery should not be considered a complication, since it is performed to ensure patient safety, conversion is associated with increase in operative time, complication rate, hospital stay length, and hospital costs^12^ ^,^ ^14^. Thus, identification of risk factors for conversion is important for better surgical planning and to avoid complications.

Several recent studies have shown that rate of LC conversion varies from 1-15%^12^ ^,^ ^23^. Conversion rate depends on the experience of the surgeon and some clinical aspects of the patients^19^ ^,^ ^23^ ^,^ ^25^. The role of gender as a risk factor for conversion from LC to open cholecystectomy and the outcome of the procedure is still debatable in the literature^26^ ^-^ ^28^. 

Similar to some reports, our study has shown that laparoscopic conversion rate to open cholecystectomy in men was similar to that of women^1,^. In addition, the rates of intraoperative complication, postoperative complication, and operative mortality were similar in both genders. Operative time and acute cholecystitis rate were the only variables evaluated that were higher in men than in women. In our series, both genders were comparable with regard to age and presence of preoperative comorbidities as determined by ASA score. 

Some authors have shown that male have higher conversion rate from LC to open cholecystectomy and operative complication rate than female^4^ ^,^ ^15^. In a recent systematic literature review, Hu et al^12^ have reported an association between some risk factors, such as older age, male gender, high body mass index, acute cholecystitis and laparoscopic conversion to open cholecystectomy. From a total of 30 studies selected by the authors, 17 have demonstrated that male gender was a risk factor for laparoscopic conversion. In this review, the most common cause of conversion was difficult dissection of Calot’s triangle during LC. A possible explanation for the higher conversion rate of LC in male is that this gender is more likely to delay seeking medical assistance and therefore present with more severe cholecystitis when they are subjected to surgical treatment^23^. 

Thesbjerg et al^26^ have reported that the main reason for higher laparoscopic conversion rate in men was due to more frequent rate of acute cholecystitis or its sequalae. Intense inflammation and firm adhesion of the gallbladder with the surrounding tissues due to cholecystitis make dissection and identification of the anatomy difficult. Bleeding during dissection further hinders safe identification of anatomy. This may impose conversion to open surgery or even cause lesion to adjacent structures. The findings of our study agree that the rate of acute cholecystitis is higher in male than in female^1^ ^,^ ^5^ ^,^ ^8^ ^,^ ^23^. In our report, the operative time was longer in male gender, possibly reflecting the difficulty in dissecting the gallbladder due to the acute cholecystitis or its sequalae. 

Surgeon’s experience is very important to reduce laparoscopic conversion and perioperative complication rates^11^. The disparity between the results of initial to recent studies is possibly due to surgeon’s greater experience and better quality of laparoscopic instruments. At the introduction of laparoscopic surgery, acute cholecystitis was a contraindication for LC. With increasing experience, the rate of laparoscopic conversion and perioperative complications has been markedly reduced. In few years, LC has become the preferable access for cholecystectomy in patients with acute inflammation of the gallbladder^23^ ^,^ ^25^. Several studies support the findings that acute cholecystitis, rather than male gender, is the most significant risk factor for laparoscopic conversion and the poor outcome of LC^1,12.16^.

Findings from some early studies on the role of gender on conversion and perioperative complication rates are limited by either small sample size or by surgeon’s experience. Although we have been performing LC since 1991, we have included in the present study only patients who underwent LC after 2011, when we had several years of experience. This may explain the reasons why our overall rate of conversion from LC to open cholecystectomy is low, even for patients with acute cholecystitis. In addition, the number of patients included in our series is high, especially if considered that all LCs were performed or supervised by a single surgeon. 

Major strengths of our study are the large sample size and few exclusion factors. All patients who were admitted to our surgical unit for either elective or emergency cholecystectomy for symptomatic gallstone were initially listed for LC. 

Limitations include the retrospective review of the data of our patients. This is minimized because all surgical procedures were coordinated and supervised by only one surgeon and the data were retrieved from electronic medical records and study protocols.

## CONCLUSION

Male gender is not an independent risk factor for laparoscopic conversion and perioperative complications.
